# Antiarrhythmic Treatment in Heart Failure

**DOI:** 10.1007/s11897-023-00642-w

**Published:** 2024-01-15

**Authors:** Hilke Könemann, Sati Güler-Eren, Christian Ellermann, Gerrit Frommeyer, Lars Eckardt

**Affiliations:** Department of Cardiology II: Electrophysiology, University Hospital Münster, Münster, Germany

**Keywords:** Heart failure, Atrial fibrillation, Ventricular arrhythmias, Sudden cardiac death

## Abstract

**Purpose of Review:**

Arrhythmias are common in patients with heart failure (HF) and are associated with a significant risk of mortality and morbidity. Optimal antiarrhythmic treatment is therefore essential. Here, we review current approaches to antiarrhythmic treatment in patients with HF.

**Recent Findings:**

In atrial fibrillation, rhythm control and ventricular rate control are accepted therapeutic strategies. In recent years, clinical trials have demonstrated a prognostic benefit of early rhythm control strategies and AF catheter ablation, especially in patients with HF with reduced ejection fraction. Prevention of sudden cardiac death with ICD therapy is essential, but optimal risk stratification is challenging. For ventricular tachycardias, recent data support early consideration of catheter ablation. Antiarrhythmic drug therapy is an adjunctive therapy in symptomatic patients but has no prognostic benefit and well-recognized (proarrhythmic) adverse effects.

**Summary:**

Antiarrhythmic therapy in HF requires a systematic, multimodal approach, starting with guideline-directed medical therapy for HF and integrating pharmacological, device, and interventional therapy.

## Introduction

Heart failure (HF) is a clinical syndrome due to structural and/or functional disorders of the heart which lead to inadequate cardiac output at rest and/or during exercise or elevated intracardiac pressures [1, 2]. Despite a decline in the age-adjusted incidence of HF in developed countries, the overall incidence of HF is increasing [3, 4]. In developed countries, the prevalence of known HF is estimated at 1–2% [5]. Globally, an estimated 64.3 million people are living with HF today [6].

Arrhythmias are frequent in patients with HF: an estimated one-third to half of all HF patients suffers from atrial fibrillation (AF) [7], and nearly half of all HF patients may have premature ventricular contractions (PVCs) [8]. At the same time, arrhythmias are associated with a significant risk of mortality and morbidity in HF patients: the combination of HF and AF may lead to higher risks for dementia, stroke, HF hospital admission, and ultimately, death [9]. Despite progress in the pharmacologic treatment of HF, the risk of sudden cardiac death (SCD) remains [10]. Especially in HF patients, SCD is often associated with ventricular arrhythmias (VA) [11, 12]. Therefore, this review focuses on antiarrhythmic long-term treatment of AF and VA in patients with HF.

## Supraventricular Arrhythmias in Heart Failure

### Atrial Fibrillation in Heart Failure

Such as HF, AF is a common cardiac condition with an approximated global prevalence of 60 million cases [13]. It is a common concept that AF begets HF and vice versa [14] as both diseases are linked by multiple risk factors for disease development and progression, e.g., arterial hypertension, diabetes mellitus, and increased age, as well as pathophysiological mechanisms. Thus, it often remains unclear if HF precedes AF or is the cause of it. HF with left ventricular systolic dysfunction can be found in more than one-third of all patients with AF and up to half of patients with HF with left ventricular systolic dysfunction suffer from AF [14, 15]. The development of AF in HF patients is associated with increased mortality in patients with both HF with preserved (HFpEF) and reduced ejection fraction (HFrEF) [16]. AF in HF patients is also associated with an increased risk of cardiovascular events in HFrEF and HFpEF patients [17]. Antiarrhythmic therapy for AF in HF patients therefore aims not only on alleviating symptoms, but early intervention is proposed to halt the progression of both HF and AF and to improve the prognosis [18, 19].

### Managing Atrial Fibrillation in Heart Failure

First, antiarrhythmic treatment of AF in HF patients includes identification and treatment of possible causes or triggers of AF [1]. This includes, e.g., hyperthyroidism, infection, and uncontrolled hypertension. All patients with AF and HF should receive guideline-directed medical therapy (GDMT) for HF [1, 19] as GDMT not only improves HF outcomes but may also affect the risk of AF. Regarding angiotensin-converting enzyme inhibitors and angiotensin receptor blockers, results of a meta-analysis by Healey et al. showed a 44% relative risk reduction in the documentation of AF in HF patients [20].

#### Rate Control

Ventricular rate control is an integral part of AF in management in all AF patients. Beta-blockers (BB) may primarily be used in patients with HFrEF and heart failure with mildly reduced ejection fraction (HFmrEF) [19, 21]. When ventricular rates remain high despite BB therapy or in case of contraindication or intolerance, digoxin should be used. Results of the DIGIT-HF trial which addresses the use of digitoxin in HFrEF patients are expected in the near future [22]. BB and digoxin may be combined if single-drug therapy does not achieve the target heart rate [19]. Of note, a combination of digitalis with class III antiarrhythmic drugs may enhance ventricular tachyarrhythmias [23].

The optimal target resting heart rate in AF patients is unclear. A lenient rate control (target resting heart rate < 110 bpm) is considered to be an acceptable approach, as data from the RACE II trial and a pooled analysis of the historic RACE and AFFIRM trials failed to show differences in outcome comparing lenient and strict rate control strategies [24, 25]. Lower ventricular rates may be targeted if required by persistent symptoms or impaired cardiac function, e.g., in case of tachycardia-induced cardiomyopathy [19].

#### Rhythm Control

The recent ESC-AF guideline gives a class I recommendation for rhythm control therapy for all symptomatic AF patients aiming at symptom control and improvement of quality of life [19]. Beyond that, the choice between a rate control strategy and a rhythm control strategy remains crucial, especially as several factors including comorbidities such as arterial hypertension and obstructive sleep apnea, left atrium enlargement, and increased sympathetic tone may complicate restoration of sinus rhythm in HF patients [26]. Early trials such as AFFIRM HF [27] and AF-CHF [28] indicated no difference between rate and rhythm control strategy regarding their endpoints of all-cause mortality and cardiovascular death in patients with concomitant AF and HF. However, as these trials were conducted in the pre-ablation era, challenges regarding optimal dosage of antiarrhythmic drugs (AAD) and their adverse effects need to be considered. Furthermore, considering the years of potential adverse effects of AF, follow-up was relatively short.

For pharmacological rhythm control, amiodarone is the drug of choice for HFrEF patients. In patients with HFpEF, amiodarone, dronedarone, or sotalol may be used [29]. Treatment with antiarrhythmic drugs for long-term rhythm control includes continuous evaluation and minimization of proarrhythmic risk [30] and organ toxicity and periodical assessment of AF burden under therapy [19].

More recent trials have provided data in favor of a rhythm control strategy.

The EAST-AFNET 4 trial [31] randomized patients with early AF (diagnosed ≤ 1 year before enrolment) and cardiovascular risk factors to early rhythm control (with AAD or catheter ablation) or usual care. 19.4% of patients received AF catheter ablation. The trial was halted prematurely because results showed a significant difference in the composite primary endpoint of cardiovascular death, stroke, or hospitalization with worsening of heart failure or acute coronary syndrome with a risk reduction of 22% by systematic rhythm control therapy. This clinical benefit was confirmed in a sub-study by Rillig et al. including patients with signs and symptoms of HF (NYHA II-III or left ventricular ejection fraction (LVEF) < 50%) [32]. Of note, the effectiveness of early rhythm control was mediated by the presence of sinus rhythm at 12 months in the EAST-AFNET 4 trial. Patients who were not in sinus rhythm at the 12-month follow-up did not further benefit from rhythm control in the remaining four years of follow-up [33]. Additionally, the clinical benefit of early rhythm control did not differ between asymptomatic and symptomatic patients in this trial [34].

The AATAC trial included patients with persistent AF, dual-chamber implanted cardioverter-defibrillator (ICD), or cardiac resynchronization (CRT) defibrillator, HF with NYHA class II-III, and LVEF ≤ 40% who were randomized to catheter ablation or amiodarone. Catheter ablation resulted in not only a significant improvement of LVEF and 6-min-walking distance, but also a relative risk reduction in hospitalization and all-cause mortality of 45% and 56%, respectively [35]. The AMICA trial failed to show a significant difference of LVEF change in HF patients with NYHA class II-III, and LVEF ≤ 35% and persistent or long-standing persistent AF comparing catheter ablation and medical treatment [36]. In addition, results of the CASTLE-AF trial (2018) [37] showed that catheter ablation for AF in patients with HF was associated with a significantly lower rate of the composite end point of death from any cause or hospitalization for worsening HF than medical therapy with an absolute risk reduction of 16.1%, suggesting prognostic effects of catheter ablation in HFrEF patients with AF. However, due to the relatively small and selected patient group with only patients with HF symptoms NYHA class ≥ II and a LVEF ≤ 35% and an implanted defibrillator, significance of the results for routine clinical practice is often doubted [38]. The CAMERA-MRI trial compared catheter ablation and medical rate control in patients with idiopathic cardiomyopathy and LVEF ≤ 45% regarding the primary endpoint of LVEF change. Results showed a significant improvement of LVEF in the ablation arm with a normalization of LVEF [39]. Furthermore, a sub-analysis of the CABANA trial for patients with concomitant AF and HF showed a reduction of the primary composite endpoint of death, disabling stroke, serious bleeding, or cardiac arrest and all-cause mortality by AF ablation compared to drug therapy including rate or rhythm control drugs [40].

A stratified pooled analysis of available trials by Chen et al. showed that catheter ablation as rhythm control strategy is associated with a significantly lower all-cause mortality, reduced re-hospitalization rate, and greater improvement in left ventricular ejection fraction compared with medical therapy in AF and HF patients [41]. Due to its publication date, results of the EAST-AFNET4 trial were not included in this analysis.

Despite several limitations [42], outcomes of these trials lead to recommendations for catheter ablation of AF as first-line therapy in the current ESC-AF guidelines when tachycardia-induced cardiomyopathy is highly probable (class I) and in selected HFrEF patients to improve survival and reduce HF hospitalization (class IIa) [43]. After failure of AAD therapy, class I recommendations for catheter ablation are given for paroxysmal and persistent AF. Beyond that, a recent AHA Scientific Statement suggests that catheter ablation may be considered first-line therapy for patients with AF and HFrEF based on this data [42]. Today, AF ablations already account for the majority of electrophysiological procedures performed in Germany [44].

For optimal patient selection for catheter ablation, several factors have been identified: in the CASTLE-AF trial, the beneficial effect of AF ablation was predominantly seen in patients with NYHA classes I–II and in patients with a nonischemic cardiomyopathy (NICM) [45]. The results of the AMICA trial [36] suggested that in patients with more advanced HF catheter ablation might not be superior to pharmacological therapy. In the CAMERA-MRI study, a greater increase in LVEF was seen in patients without left ventricular scar determined by late gadolinium enhancement on cardiac MRI [39]. LV scarring on MRI might represent advanced cardiomyopathy often concomitant with increased left atrial volume and diameter and persistent AF [42]. While patients with end-stage heart failure were excluded from aforementioned studies, the recently published CASTLE-HTx trial [46] aimed to evaluate the effect of catheter ablation of AF in this specific cohort of patients with end-stage HF who are eligible of heart transplantation. The results of this trial showed a reduction in the composite endpoint consisting of death from any cause, left ventricular assist device implantation, or urgent heart transplantation with the combination of catheter ablation of AF and guideline-directed medical therapy compared to medical therapy alone after a median follow-up of 18 months in this patient cohort. The trial was stopped early for efficacy on the recommendation of the monitoring board. Although the open-label design, the relatively small sample size, and the single-center nature of the study are important limitations, the results of CASTLE-HTx point towards a benefit of AF ablation in patients with the most advanced HF.

**Fig. 1:**
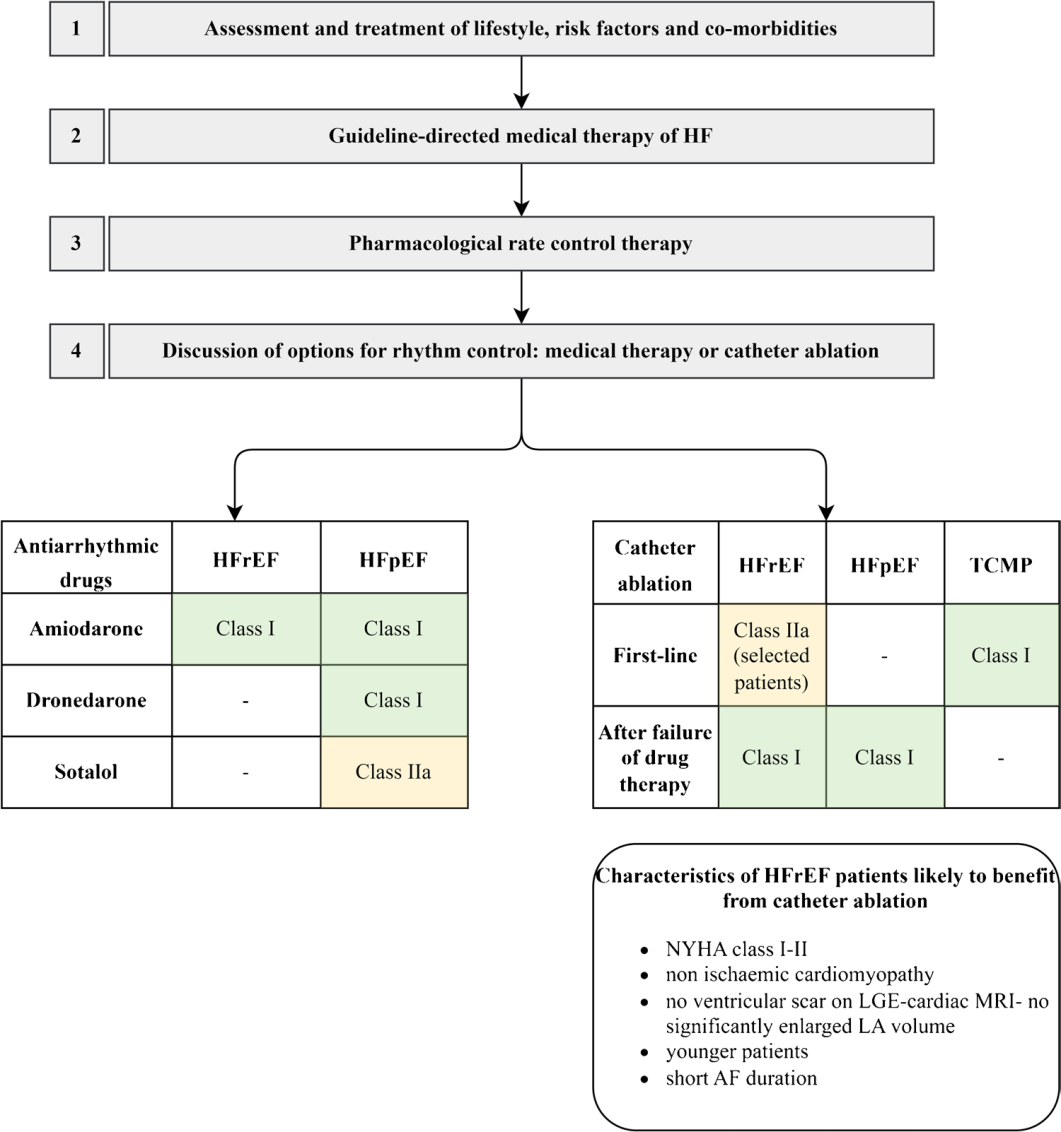
Schematic overview on therapy of atrial fibrillation in heart failure patients

In general, a shared decision-making process is recommended, considering patient preferences as well as several factors regarding HF and AF symptoms, LVEF impairment, left ventricular scarring, and duration of AF (Fig. 1).

#### Atrioventricular Node Ablation and Pacing/CRT

Pharmacological ventricular rate control can be challenging in HF patients. In a systematic review by Ganesan et al. [44] atrioventricular node ablation in HF patients with AF was associated with a significant reduction in all-cause mortality, cardiovascular mortality, and improvement in mean NYHA class [47]. More recently, the APAF-CRT trial showed that ablation of the atrioventricular node with prior implantation of CRT was superior to conventional pharmacological rate control due to significant reduction in death resulting from HF and HF hospitalisations in patients with permanent AF and at least one hospitalization for HF in the prior year [48]. Based on these data, atrioventricular node ablation [49] and CRT implantation may be preferred when a rate control strategy is pursued.

### Other Types of Supraventricular Arrhythmias

HF patients can develop paroxysmal supraventricular arrhythmias that are otherwise seen in the healthy population, e.g., atrioventricular nodal re-entrant tachycardia, atrioventricular re-entrant tachycardia, or focal atrial tachycardia. Dependent on type, rate, and duration of tachycardia, they may cause arrhythmia-induced or arrhythmia-aggravated cardiomyopathy [50]. Generally, these arrhythmias are treated similarly to patients without HF, although the main treatment goal is the elimination of tachycardia either by AAD and/or catheter ablation. Therefore, the threshold for catheter ablation should be lower in HF patients with arrhythmias known to have a high success rate of catheter ablation [50, 51].

## Ventricular Arrhythmias in Heart Failure

### Ventricular Arrhythmias and Sudden Cardiac Death

Triggered by a pivotal meta-analysis of randomized controlled trials (RCTs) by Shen et al. [52] including 40,195 patients, there has been increasing attention recently to the notion that the risk of SCD in patients with HF is reduced with GDMT. Nevertheless, there is no convincing evidence for a significantly declined SCD risk in HF trials over time [10]. SCD still is a very common cause of death in patients with HF. In a meta-analysis including 1.5 million patients with HF, Jones et al. [53] showed that SCD accounted for 22% of all deaths from 2007 to 2017 with no apparent reduction over time. Furthermore, despite available data suggests that GDMT reduces the relative risk of SCD [52], even in recent HF trials [54, 55], the annual risk of SCD remains higher than 1.2% [10].

Several studies have shown that a high proportion of deaths in HF patients occurs suddenly as a result of VA [56, 57]. In a recent population-based cohort study, the 1-year cumulative incidence of severe VA defined as VA associated with emergency department visits or hospitalizations was 5.4% in patients with advanced HF with LVEF < 40% [58]. New-onset VA were associated with increased mortality. Thus, optimal treatment of VA in patients with HF includes primary and secondary preventive ICD therapy as well as pharmacological and interventional treatment of VA.

#### Pharmacological Treatment

GDMT is the basis of antiarrhythmic treatment of HF patients in order to prevent progression of the underlying cardiomyopathy. This should include angiotensin-converting enzyme inhibitors (ACE-I) or angiotensin receptor-neprilysin inhibitors (ARNI), BB, mineralocorticoid receptor antagonists (MRA), and sodium glucose co-transporter 2 inhibitors (SGLT2-I) in HFrEF patients (all class I recommendations) [1]. As prospective RCTs for patients specifically with HFmrEF and HFpEF are lacking recommendations for these patients, groups are weaker and emphasize the use of diuretics, when needed, and SGLT2-I [1, 2]. With regard to BB, MRA, and ARNI, multiple studies have shown a relative risk reduction of SCD [10], e.g., bisoprolol reduced SCD by 44% in the CIBIS II study [59] and ARNI reduced SCD risk by 20% in the PARADIGM-HF trial [60].

Until today, no AAD except for BB has proven to reduce all-cause mortality. Nevertheless, AAD remain integral part of the management of VA in HF patients as adjunctive therapy, especially for symptomatic patients with frequent VA. Yet, besides proarrhythmic [30] and other drug-specific adverse effects, most AAD have negative-inotropic effects that may worsen the hemodynamic status. Class IC AAD are generally avoided in patients with structural heart disease and impairment of LVEF due to the results of the CAST trial [61]. Similarly, an increased mortality has been shown for dronedarone [62] and sotalol [63]; thus, amiodarone is the most-widely used AAD in HF patients as it has shown neutral effects on mortality in clinical trials of patient with HFrEF [2] and demonstrates a low proarrhythmic potential in HF [64, 65]. Amiodarone is also the drug of choice in case of an electrical storm [66]. Amiodarone combined with BB has shown a high efficacy regarding ICD shock rates [67] which has to be weighed against the increased rate of adverse events, e.g., thyroid and pulmonary toxicity [66]. Class IB AAD such as lidocaine or mexiletine and Class IA AAD as quinidine have not been studied systematically and may be used for refractory arrhythmias after individual risk–benefit assessment [66].

#### Device Therapy

ICD therapy is essential in secondary prevention of SCD in patients with a history of aborted SCD or hemodynamically significant sustained VA based on the results of relatively old ICD trials [66, 68–70]. For primary prevention of SCD, several RCTs [71–74] support ICD therapy in HF patients with LVEF ≤ 35% by reporting a significant mortality reduction in this patient cohort. As the evidence is most robust in patients with ischemic etiology of HF, the current ESC guideline on VA and prevention of SCD gives a strong class I recommendation for symptomatic HFrEF patients with NYHA functional class II–III and LVEF ≤ 35% [66, 75]. Mainly due to the more recent DANISH trial [76] that raised questions on the benefit of ICD therapy in patients with NICM as it failed to show a reduction in the primary endpoint of all-cause death by ICD therapy compared to standard care, the current ESC guideline gives a class IIa recommendation for this patient group. Yet, in a meta-analysis, a significant reduction of overall mortality was shown by primary preventive ICD therapy in patients with NICM [77].

All in all, as the absolute majority of SCD cases occurs in patients with HFmrEF or HFpEF [56], the significance of LVEF as the only risk marker is limited, especially in NICM patients. The current ESC guidelines [66] have addressed this concern by considering additional risk factors such as specific pathogenetic mutations, history of syncope, inducibility of sustained monomorphic ventricular tachycardia (VT) on electrophysiological study, and increased scar burden on LGE cardiac MRI [78]. Additionally, echocardiographic variables may assist assessing the prognosis in HF patients [79]. Regarding primary preventive ICD therapy, GDMT for HF is required for 3 months until the decision for ICD implantation is made [66]. 

Table 1 provides an overview of indications on ICD implantation according to current ESC [1] guidelines. Notably, due to publication dates and different weighing of the available evidence, recommendations for primary preventive ICD therapy in HF patients differ internationally [80].

**Table 1:** Overview on recommendations on primary preventive ICD therapy in patients with stable coronary artery disease and non-ischemic and dilated cardiomyopathy according to current ESC HF [1] and VA/SCD [66] guidelines

Coronary artery disease	ESC guidelines recommendation
LVEF^a^ ≤ 35% + NHYA^c^ class II–III despite ≥ 3 months of OMT^d^	Class I
LVEF^a^ ≤ 35% + NHYA^c^ class I despite ≥ 3 months of OMT^d^	Class IIa
LVEF^a^ ≤ 40% despite ≥ 3 months of OMT^d^ + nsVT^b^ + inducible monomorphic VT^e^	Class IIa
LVEF^a^ ≤ 40% despite ≥ 3 months of OMT^d^ + unexplained syncope + inducible monomorphic VT^f^	Class IIa
NHYA^c^ class IV candidates for cardiac transplantation	Class IIa
Within 40 days of myocardial infarction	Class III
Non-ischemic cardiomyopathy	
LVEF^a^ ≤ 35% + NHYA^c^ class II–II despite ≥ 3 months of OMT^d^	Class IIa
Dilated cardiomyopathy	
Pathogenic mutation in *LMNA* gene and estimated 5-year risk of VA^e^ ≥ 10% + nsVT^b^ or LVEF^a^ < 50% or atrioventricular conduction delay	Class IIa

^a^*LVEF*, left ventricular ejection fraction; ^b^*nsVT*, non-sustained ventricular tachycardia; ^c^*NYHA*, New York Heart Association; ^d^*OMT*, optimal medical therapy; ^e^*VA*, ventricular arrhythmia; ^f^*VT*, ventricular tachycardia

About one-third of all HF patients has ventricular conduction abnormalities [50, 81]. In selected HF patients, cardiac resynchronization therapy (CRT) reduces morbidity and mortality [1, 82, 83]. Therefore, according to current guidelines, selected HFrEF patients with an indication for ICD therapy may receive CRT-ICD rather than conventional ICD therapy. The strongest recommendation (class I) is given for symptomatic HF patients with LVEF ≤ 35% in sinus rhythm who show a left bundle branch QRS morphology with a QRS duration ≥ 150 ms, while a class IIa recommendation is given for patients with a QRS duration ≥ 150 ms but non-left bundle branch QRS morphology [1, 83].

#### Catheter Ablation

ICD therapy reduces SCD, but does not prevent VT. Therefore, many HF patients may experience symptomatic VA and ICD shocks. Catheter ablation of VA is therefore a central component of VA therapy in HF patients. In the VANISH trial, there was a significant reduction of the composite endpoint of death, VT storm, or appropriate ICD therapy in the ablation group as compared to the group receiving an escalation in pre-existing AAD therapy [84]. The trial only included patients with ischemic cardiomyopathy with a mean LVEF of 31%. Results of the BERLIN VT trial failed to show a reduction in mortality or hospitalization for arrhythmia or worsening HF during 1 year of follow-up by preventive ablation immediately before ICD implantation compared to a deferred ablation therapy after the third appropriate ICD shock [85]. At the moment, the ongoing VANISH2 trial (NCT02830360) is aiming at addressing the question whether catheter ablation is superior to AAD therapy as first-line treatment. Overall, optimal timing of catheter ablation of VT is unclear [86] and data on a possible prognostic benefit of catheter ablation are scarce. The recently published SURVIVE-VT trial [87] and the PARTITA trial [88] provide evidence for earlier consideration of VT ablation in clinical practice after a first appropriate ICD shock [89]. Furthermore, the PAUSE-SCD trial [90] reported a reduction of the composite endpoint of VT recurrence, cardiovascular hospitalization, and death by early ablation performed at the time of ICD implantation in ischemic and NICM. A recent meta-analysis [91] of nine RCTs comparing the efficacy of early VT catheter ablation demonstrated that early ablation reduces VT burden and ICD therapies. However, mortality rate and quality of life were not affected. As only 7.9% of included patients had a nonischemic etiology of HF, RCTs on catheter ablation of VT in this patient group are needed to clarify the role of ablation in this cohort. Thus, catheter ablation was particularly recommended in HF patients with ischemic cardiomyopathy and recurrent ventricular tachycardia after ICD therapy.

### Premature Ventricular Contractions and PVC-aggravated Cardiomyopathy

PVCs are the most frequent VA [50] and common in patients with HF. Frequent PVC may cause left ventricular systolic dysfunction referred to as PVC-induced or PVC-aggravated cardiomyopathy. As cardiomyopathy may be reversed by the elimination of PVCs, it is important to recognize this entity. However, despite elimination of PVCs, in some patients, LVEF does not return to normal, which may be caused by a preexisting (yet unknown) left ventricular dysfunction [75]. Thus, the diagnosis of PVC-induced cardiomyopathy can only be confirmed after improvement or normalization of LVEF following elimination of PVCs. A PVC burden of > 10% seems to be the threshold of PVCs for the development of left ventricular dysfunction [92].

Treatment of PCV-mediated cardiomyopathy therefore aims at complete suppression of PVCs. Catheter ablation of PVCs has reported success rates of 75–90% [66] and is therefore considered first-line treatment (class I recommendation) for PVC-induced cardiomyopathy. PVC ablation can be challenging [93, 94] due to catheter instability or the inability to reach PVC origin, especially when an intramural location is present [50]. AAD are an alternative if catheter ablation is not desired, suspected to be high-risk, or unsuccessful. When a PVC-induced cardiomyopathy without other underlying structural heart disease is suspected and there is only a moderate left ventricular dysfunction, flecainide can be used apart from BB and amiodarone.

## Conclusion

**Fig. 2:**
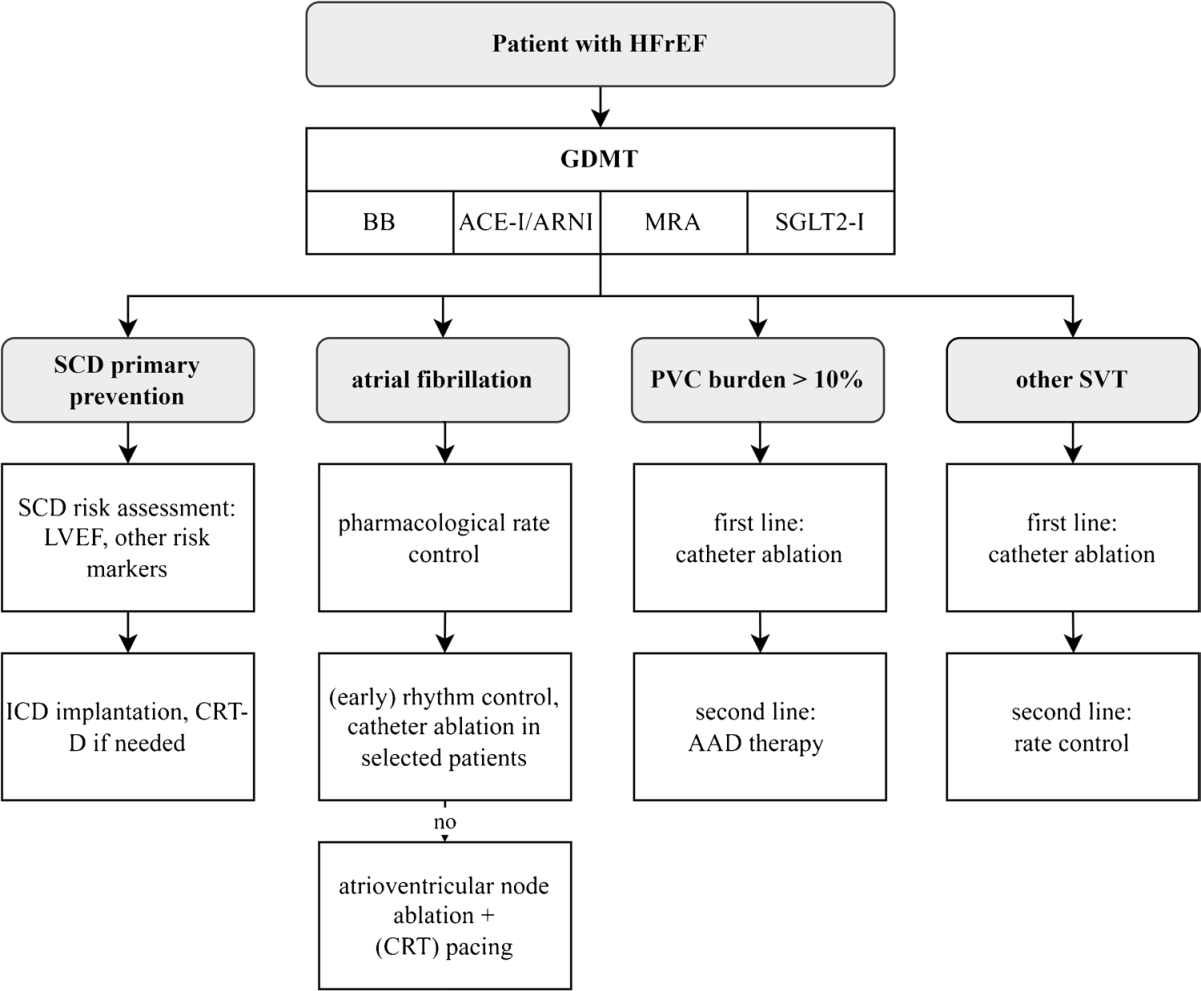
Schematic representation of judgement that is recommended for optimal antiarrhythmic treatment of heart failure patients as presented in this article

The management of arrhythmias in HF requires a systematic, multimodality approach. It starts with GDMT for HF as the foundation and should integrate pharmacological, interventional, and device therapy for arrhythmias (Fig. 2). AF and HF are often linked together. For the treatment of catheter ablation, all patients may receive pharmacological rhythm control therapy. In HFrEF patients, (early) rhythm control for AF including catheter ablation is an important pillar of AF therapy, as recent RCTs showed a reduction of AF burden and suggested prognostic implications, especially in case of arrhythmia-induced cardiomyopathy. On the other hand, data for AF therapy in patients with HFpEF are sparse. When a rate control strategy is chosen over rhythm control, atrioventricular node ablation and (CRT) pacing might be considered early. The treatment of other supraventricular arrhythmias is similar to the management in patients without HF, but due to the possibility to tachycardia-induced cardiomyopathy, thresholds for catheter ablation as a curative therapy should be lower.

Despite a reduction of relative SCD risk by a progress in pharmacological HF therapy, absolute SCD risk remains high in HF patients and SCD is often associated with VA. ICD therapy is crucial not only in secondary, but primary prevention of SCD risk. For risk stratification, LVEF ≤ 35% is used as a primary risk factor based on older primary preventive ICD trials. Nevertheless, a shift to a more personalized assessment of SCD risk in the individual patient integrates other factors such as LGE on cardiac MRI, specific pathogenic mutations and electrophysiologic study can be observed, especially in patient with NICM. In patients with an indication for ICD and additional intraventricular conduction disturbances, CRT should be considered according to current guidelines. Until now, AAD failed to show a positive effect on mortality in HF patients, except for BB. Yet, AAD are important as adjunct therapy in patients with frequent and symptomatic VA. Amiodarone is often the AAD of choice, because class IC AAD and sotalol have been shown to increase mortality in patients with structural heart disease and dronedarone has been associated with increased early mortality in patients with severe HF. Catheter ablation of ventricular tachycardia is another important strategy with the potential to decrease the number of (symptomatic) VT recurrences in HF patients. Recent RCTs provide support for the consideration of early VT ablation in clinical practice, although available data is most robust for patients with ICM. In HF patients with PVC-induced or PVC-aggravated cardiomyopathy, catheter ablation of PVC is considered as first-line treatment due to high success rates, although this treatment option can be challenging depending on the site of origin of the arrhythmia.
